# The evolving epidemiology of Carbapenemase-producing *Enterobacterales* in Canadian acute care facilities, 2010–2023

**DOI:** 10.1186/s13756-025-01602-w

**Published:** 2025-07-12

**Authors:** Robyn Mitchell, Laura Mataseje, Joëlle Cayen, Erin McGill, Kristine Cannon, Ian Davis, Tamara Duncombe, Chelsey Ellis, Jennifer Ellison, Jennifer Happe, Susy S. Hota, Kevin C. Katz, Pamela Kibsey, Santina Lee, Jerome A. Leis, Xena Li, Allison McGeer, Jessica Minion, Sonja Musto, Connie Patterson, Ewa Rajda, Stephanie W. Smith, Jocelyn A. Srigley, Kathryn N. Suh, Nisha Thampi, Jen Tomlinson, Joseph Vayalumkal, Kristen Versluys, Titus Wong, Yves Longtin

**Affiliations:** 1 Public Health Agency of Canada, 130 Colonnade Road, Ottawa, ON K1A 0K9 Canada; 2 National Microbiology Laboratory, Public Health Agency of Canada, Winnipeg, MB Canada; 3 Alberta Health Services, Calgary, AB Canada; 4 Dalhousie University, Halifax, NS Canada; 5 Fraser Health Authority, Surrey, BC Canada; 6 The Moncton Hospital, Moncton, NB Canada; 7 University Health Network, Toronto, ON Canada; 8 North York General Hospital, Toronto, ON Canada; 9 Vancouver Island Health Authority, Victoria, BC Canada; 10 Health Sciences Centre, Winnipeg, MB Canada; 11 Sunnybrook Health Sciences Centre, Toronto, ON Canada; 12 Sinai Health System, Toronto, ON Canada; 13 Saskatchewan Health Authority, Regina, SK Canada; 14 McGill University Health Centre, Montréal, QC Canada; 15 University of Alberta, Edmonton, AB Canada; 16 BC Children’s & Women’s Hospitals, Vancouver, BC Canada; 17 The Ottawa Hospital, Ottawa, ON Canada; 18 Children’s Hospital of Eastern Ontario, Ottawa, Canada; 19 Alberta Children’s Hospital, Calgary, AB Canada; 20 Interior Health, Kelowna, BC Canada; 21 Provincial Health Services Authority, Vancouver, BC Canada; 22 Jewish General Hospital, Montréal, QC Canada

**Keywords:** Carbapenemase-producing *Enterobacterales*, Epidemiology, Surveillance, Nosocomial transmission, Infection prevention

## Abstract

**Background:**

Carbapenemase-producing *Enterobacterales* (CPE) are associated with substantial morbidity and mortality with limited treatment options and have an ability to spread rapidly in healthcare settings. We analyzed surveillance data from the Canadian Nosocomial Infection Surveillance Program to describe trends and the epidemiology of CPE from 2010 to 2023.

**Methods:**

Participating acute-care hospitals submitted eligible isolates to the National Microbiology Laboratory for detection of carbapenemase genes. Trained infection control professionals applied standardized definitions to collect epidemiological data by chart review from 30–97 hospitals from 2010 to 2023.

**Results:**

The national incidence of CPE infection (0.03 to 0.14 per 10,000 patient days; R^2^ = 0.76) and colonization (0.02 to 0.78 per 10,000 patient days; R^2^ = 0.83) increased exponentially from 2010 to 2023. We identified rapidly rising rates of healthcare-associated (HA) CPE infections from 2019 to 2023 (0.05 to 0.09 per 10,000 patient-days, *p* = 0.04), attributed to select hospitals (7/97) which accounted for half (53%) of all HA-CPE infections in 2023. Similarly, we identified that 2023 HA-CPE colonization rates were highest in medium (201–499 beds) and large (≥500 beds) hospitals in the Central region.

Most patients did not report international travel (66%) nor receipt of medical care abroad (74%). Travel and receipt of medical care were less commonly reported among *bla*_KPC_ associated cases (7.1% and 5.3% respectively) compared to *bla*_NDM_ (55% and 45% respectively) and *bla*_OXA-48_ (57% and 39%) associated cases. Furthermore, *bla*_KPC_ was the predominant carbapenemase among all HA-CPE isolates (62%, 950/1,534).

**Conclusions:**

Surveillance data from a national network of Canadian acute care hospitals indicates that while the incidence of CPE in Canada remains low, it is accelerating at an exponential rate. Our findings suggest that nosocomial transmission is driving the recent increase in CPE incidence in Canada. Improved infection control measures and antimicrobial stewardship as well as access to newer antimicrobials are all urgently needed.

**Supplementary Information:**

The online version contains supplementary material available at 10.1186/s13756-025-01602-w.

## Background

Carbapenemase-producing *Enterobacterales* (CPE) have rapidly become a global health concern. They are associated with substantial morbidity and mortality with limited treatment options [1–4]. CPE is predominantly a healthcare-associated pathogen and major routes of transmission include direct or indirect patient-to-patient transmission, environmental contamination, contaminated hands and medical devices, as well as interfacility transmission [2, 5–8]. As a result, the control of CPE has become an increasing challenge for healthcare facilities and monitoring the incidence and epidemiology of CPE has become critical to informing infection prevention and control efforts to limit transmission.

The incidence of CPE varies globally, ranging from sporadic imported cases and hospital outbreaks to regional endemicity [1, 2]. However, a common trend among several countries is an increase in the incidence and transmission of CPE [9]. Regionally, Nordic countries including Norway, Switzerland, Finland and Denmark have reported a low yet increasing incidence of CPE resulting in outbreaks and clusters [10–13]. The Korean Centre for Disease Prevention and Control as well as Australia’s National Alert System for Critical Antimicrobial Resistance have reported rapid increases in CPE [14, 15]. The results of national surveillance in Italy showed a high burden of CPE bloodstream infections [16] aligned with concerning trends of rising rates of invasive carbapenem-resistant gram-negative bacterial infections reported to the European Antimicrobial Resistance Surveillance Network [17].

Previously in Canada, CPE was initially limited to individual cases and clusters identified in a few hospitals [18–21]. Between 2010 and 2014, Canadian Nosocomial Infection Surveillance Program (CNISP) national surveillance data found no significant increase in the incidence of CPE infected and colonized patients [22]. However, in recent years, an increasing trend in the incidence of CPE has been reported regionally and nationally in Canada [23–26].

To better understand the current Canadian situation and inform prevention and control measures, we describe trends and the epidemiology of CPE among acute-care hospitals participating in the CNISP.

## Methods

### Sources of data and study population

CNISP is a collaboration between the Public Health Agency of Canada (PHAC), including the National Microbiology Laboratory (NML), the Association of Medical Microbiology and Infectious Disease Canada, and sentinel hospitals across Canada [27]. In 2023, the CNISP network included 106 acute care hospitals located in all 10 provinces and one territory, totalling approximately 28,724 adult and pediatric inpatient beds (37% of total national capacity) and 1,307,500 annual inpatient admissions.

All *Enterobacterales* collected from a patient (colonized or infected) admitted to a CNISP participating hospital, emergency department or outpatient clinic that exhibited nonsusceptibility to imipenem, meropenem or ertapenem in accordance with the Clinical and Laboratory Standards Institute (CLSI M100-ED34:2024) or if they tested positive using molecular PCR testing for carbapenemase genes or phenotypic testing (ex. mCIM, CARBA-NP, NG-test® CARBA 5) were considered eligible for inclusion and confirmation. All eligible isolates were submitted to the NML for detection of the following carbapenemase genes (NDM, OXA-48-like, KPC, IMP, VIM, GES, SME, NMC/IMI) [25, 28]. Only cases that were positive for one of the above carbapenemase targets were included in this analysis.

### Data collection and case definitions

Epidemiologic (demographic, clinical, risk factor, and outcome) and denominator data (patient-days) were collected by trained infection control professionals by chart review and submitted electronically to PHAC using a standardized protocol [28] and data collection form (Supplemental table S1). Cases were defined as patients admitted to a CNISP participating hospital between January 1, 2010, and December 31, 2023, with carbapenemase-producing *Enterobacterales* isolated from a screening or clinical specimen. Patients identified in the emergency department and outpatients were excluded from this analysis. Isolate data were linked to epidemiological data using an anonymized unique patient identifier. The surveillance period was based on the date of positive culture. Duplicate cases were removed as only the first CPE species/carbapenemase combination per patient was included per surveillance year. (e.g. *K. pneumonia* KPC and *K. pneumonia* NDM from the same patient in the same surveillance year would be included). The microbiological results represent all eligible isolates. In the epidemiological results, for isolates isolated simultaneously from multiple sites, only those from the most clinically pertinent sites were retained (e.g. blood cultures override urine cultures). If multiple isolates were recovered from the same site, only the first isolate was included in the analysis.

Travel history was defined as international travel in the 12 months prior to positive culture. Data on origin of acquisition were collected beginning in 2015. Inpatients with a positive culture collected on or beyond calendar day 3 (48 h) of their hospitalization, or who had a healthcare exposure in Canada which may have resulted in their infection or colonization, were classified as healthcare-associated (HA). If the healthcare exposure occurred outside of Canada, the case was classified as HA (foreign healthcare exposure). If the patient did not have any exposure to healthcare that would have resulted in their infection or colonization (using best clinical judgement) and did not meet the criteria for a HA infection or colonization, the case was classified as community-associated. Cases where the source of acquisition could not be determined were excluded from the acquisition analyses. Clinical infections were defined as CPE positive patients with signs and symptoms and were categorized using the National Healthcare Safety Network definitions [29] whereas colonizations were defined as a clinical sample positive for CPE without signs and symptoms. Thirty-day all-cause mortality and intensive care unit (ICU) admission were collected for patients with clinical infections only.

### Statistical analysis

Patient characteristics and outcomes were described. We reported continuous variables as medians with interquartile ranges (IQRs) and categorical variables as absolute values and percentages. Chi-squared or Fisher’s exact tests were used to compare proportions and the Kruskal–Wallis rank sum test used to compare medians. Missing and incomplete data for individual variables were excluded from analyses, therefore denominators may vary. Provinces were grouped into three regions: west (British Columbia, Alberta, Saskatchewan, and Manitoba), central (Ontario and Québec) and east (Nova Scotia, New Brunswick, Prince Edward Island and Newfoundland and Labrador).

Annual CPE infection and colonization incidence rates were calculated per 10,000 patient-days. The Mann–Kendall test was used to test trends. We fit simple linear regression and exponential regression growth models to CPE infection and colonization rates. Analyses were conducted using R statistical software version 4.3.2 [30].

## Results

### CPE infection and colonization results

From 2010 to 2023, 2,331 (81%) colonized and 539 (19%) infected CPE patients were reported. Participating hospitals increased from 30 in 2010 to 97 in 2023. The characteristics of participating hospitals in 2010 were compared to those participating in 2023 (Supplemental Table S2). The changes in hospitals characteristics are reflective of the addition of small, community hospitals as well as those from under-represented regions to improve the representativeness of the hospital network. Table 1 describes the patient characteristics and 30-day outcomes. The median age of all CPE patients was 67 years (IQR 54–77 years), and infected patients were younger compared to colonized patients (65 years vs. 67 years, *p* < 0.001). Few cases (*n* = 95, 3.3%) were reported among pediatric patients (< 18 years). Pre-existing comorbidities were common (84%) and included heart disease (32%), diabetes (29%), liver disease (7.8%), active cancer (7.7%) and kidney disease (7.5%).

**Table 1:** Selected characteristics and outcomes of CPE colonized and infected inpatients, 2010–2023

Characteristic	Overall *N* = 2870	Colonized patients *N* = 2331	Infected patients *N* = 539	*P*-value^*^
**Sex, male**	1,667/2,836 (59%)	1,364/2,311 (59%)	303/525 (58%)	0.58
**Age, years**				
Median (IQR)	67 (54, 77)	67 (55, 78)	65 (51, 73)	** < 0.001**
**Age group**				0.14
Adult	2,747/2,842 (97%)	2,245/2,317 (97%)	502/525 (96%)	
Pediatric	95/2,842 (3.3%)	72/2,317 (3.1%)	23/525 (4.4%)	
**Pre-existing comorbidities**	1,974/2,337 (84%)	1,542/1,845 (84%)	432/492 (88%)	**0.021**
Heart disease	736/2,334 (32%)	624/1,844 (34%)	112/490 (23%)	** < 0.001**
Diabetes	687/2,335 (29%)	532/1,844 (29%)	155/491 (32%)	0.24
Liver disease	182/2,328 (7.8%)	140/1,839 (7.6%)	42/489 (8.6%)	0.47
Cancer (active)	180/2,336 (7.7%)	130/1,845 (7.0%)	50/491 (10%)	**0.021**
Kidney disease	175/2,336 (7.5%)	132/1,845 (7.2%)	43/491 (8.8%)	0.23
Lung disease	121/2,336 (5.2%)	96/1,845 (5.2%)	25/491 (5.1%)	0.92
SOT recipient	111/2,335 (4.8%)	74/1,844 (4.0%)	37/491 (7.5%)	**0.001**
BMT recipient	39/2,336 (1.7%)	22/1,845 (1.2%)	17/491 (3.5%)	** < 0.001**
Other immunosuppression	24/1,139 (2.1%)	18/888 (2.0%)	6/251 (2.4%)	0.72
HIV infection	17/2,336 (0.7%)	16/1,845 (0.9%)	1/491 (0.2%)	0.23
Other condition	648/2,338 (28%)	478/1,846 (26%)	170/492 (35%)	** < 0.001**
**Ward at time of positive culture**				
Medical ward	1,412/2,735 (52%)	1,199/2,225 (54%)	213/510 (42%)	** < 0.001**
Surgical ward	610/2,735 (22%)	508/2,225 (23%)	102/510 (20%)	0.17
ICU	527/2,735 (19%)	388/2,225 (17%)	139/510 (27%)	** < 0.001**
Transplant/BMT/ Hematology/Oncology	53/2,735 (1.9%)	28/2,225 (1.3%)	25/510 (4.9%)	** < 0.001**
Other ward^†^	133/2,735 (4.9%)	102/2,225 (4.6%)	31/510 (6.1%)	**0.016**
**Site of isolation**				
Stool/rectal swab	2,049/2,849 (72%)	2,049/2,316 (88%)	0/533 (0%)	NA
Urine	348/2,849 (12%)	150/2,316 (6.5%)	198/533 (37%)	** < 0.001**
Blood	147/2,849 (5.2%)	0/2,316 (0%)	147/533 (28%)	NA
Respiratory	112/2,849 (3.9%)	47/2,316 (2.0%)	65/533 (12%)	** < 0.001**
Wound	70/2,849 (2.5%)	25/2,316 (1.1%)	45/533 (8.4%)	** < 0.001**
Skin/soft tissue	36/2,849 (1.3%)	16/2,316 (0.7%)	20/533 (3.8%)	** < 0.001**
Surgical site	16/2,849 (0.6%)	1/2,316 (< 0.1%)	15/533 (2.8%)	** < 0.001**
Other	71/2,849 (2.5%)	28/2,316 (1.2%)	43/533 (8.1%)	** < 0.001**
**Acquisition**				
HA (Canada)	1,746/2,646 (66%)	1,438/2,170 (66%)	308/476 (65%)	0.51
HA (Foreign healthcare exposure)	457/2,646 (17%)	386/2,170 (18%)	71/476 (15%)	0.13
Community-associated	262/2,646 (9.9%)	219/2,170 (10%)	43/476 (9.0%)	0.48
Unable to determine	181/2,646 (6.8%)	127/2,170 (5.9%)	54/476 (11%)	** < 0.001**
**Travel**	725/2,143 (34%)	598/1,717 (35%)	127/426 (30%)	0.050
**Medical care abroad**	528/2,065 (26%)	432/1,650 (26%)	96/415 (23%)	0.20
**30-day outcomes among infected patients only**				
**30-day ICU admission**	46/383 (12%)	NA	46/383 (12%)	NA
**30-day outcome**				
Patient alive, still in hospital	164/505 (32%)	NA	164/505 (32%)	NA
Patient survived and transferred	31/505 (6.1%)	NA	31/505 (6.1%)	NA
Patient survived and discharged	216/505 (43%)	NA	216/505 (43%)	NA
Patient died	94/505 (19%)	NA	94/505 (19%)	N/A

Missing and incomplete data for individual variables were excluded from analyses, therefore denominators may vary

*IQR* Interquartile range, *ICU* Intensive care unit, *BMT* Bone marrow transplant, Respiratory include sputum, endotracheal secretions and bronchoalveolar lavage, *SOT* Solid organ transplant, *HA* Healthcare-associated, *NA* Not applicable

^*^p values reported for comparison between infected and colonized patients. Bold type indicates statistical significance (*p* < 0.05)

^†^Other wards include: hemodialysis, pediatrics, obstetrics/gynecology, neonatal intensive care unit, pediatric intensive care unit, rehabilitation and orthopedics etc

Among CPE infected patients, urine (37%), blood (28%) and lower respiratory tract (sputum, endotracheal secretions, bronchoalveolar lavage) (12%) were the most common positive specimen types identified from 2010 to 2023.

From 2010 to 2023, 34% (725/2,143) of patients with CPE reported international travel in the 12 months prior to positive culture. The majority of them reported travel to South Asia (56%, 374/664) followed by Africa (11%, 74/664), South America (11%, 74/664), Asia (8%, 55/664), Europe (7%, 48/664), the Middle East (4%, 23/664) and North America (2%, 16/664). The proportion of patients reporting travel was stable from 2016 (35%, 33/94) to 2023 (37%, 252/685) *p* = 0.8, however a decrease in travel was identified in 2021 (20%, 39/198), most likely related to COVID-19 pandemic travel restrictions. From 2010 to 2023, 26% (528/2,065) of CPE patients received medical care while abroad. Similarly, the proportion of positive patients who received medical care while abroad was stable from 2016 (28%, 26/92) to 2023 (26%, 166/649) *p* = 0.7, except for a decrease in 2021 (17%, 34/197).

Among infected patients, 12% (46/383) were admitted to an intensive care unit within 30 days of a positive culture. Thirty-day all-cause mortality was 19% (94/505) for all infected patients and 30% (41/135) for patients with bacteremia. Among infected inpatients who died, preexisting comorbidities were common (90%, 77/86) and included diabetes (32%), heart disease (26%), liver disease (18%), active cancer (12%) and kidney disease (10%).

### CPE infection results

**Fig. 1:**
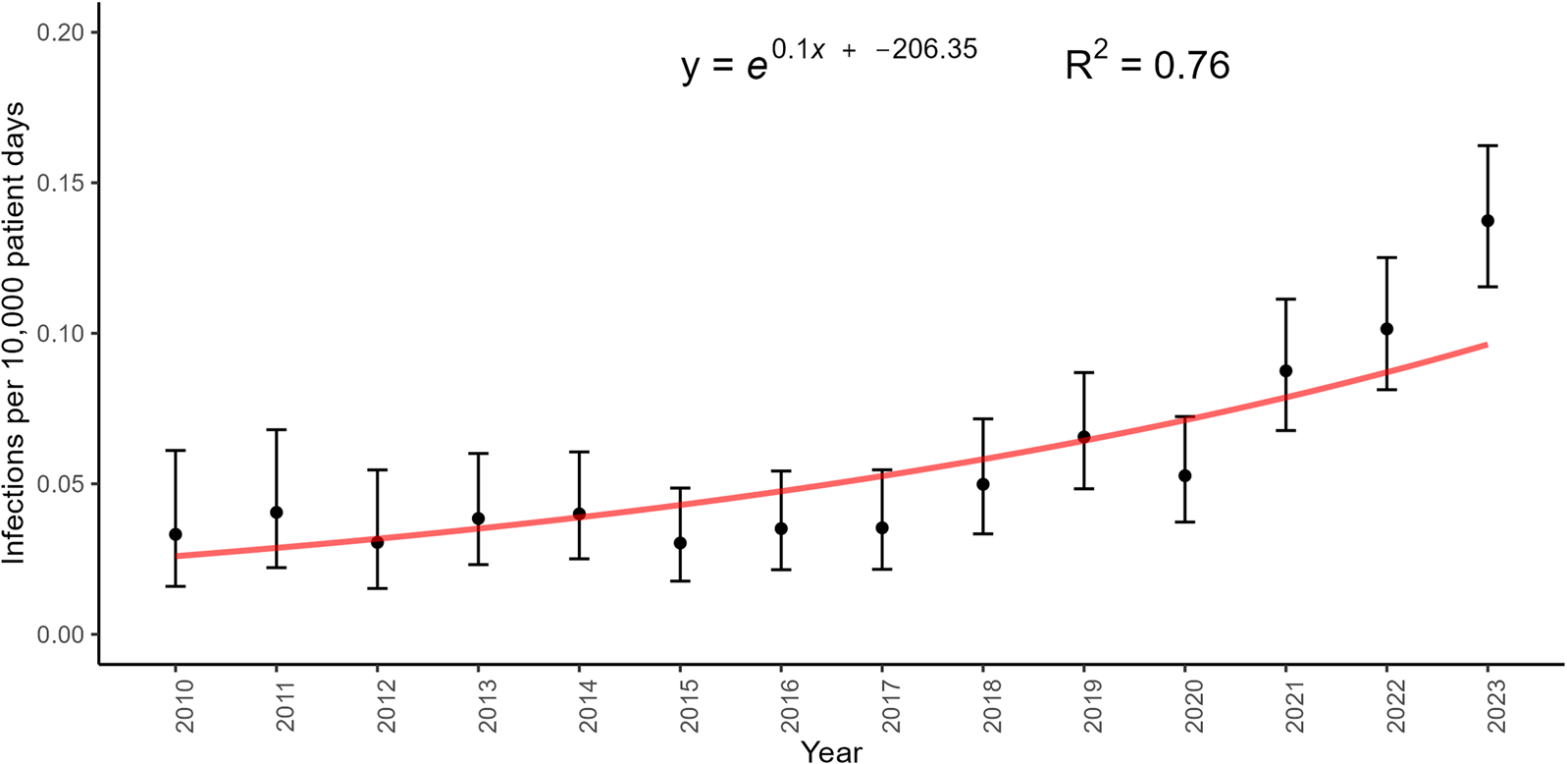
Incidence of national CPE infection rates with 95% confidence intervals and expressed as an exponential equation, 2010–2023

The incidence of CPE infections in CNISP participating hospitals remains low with 138 infections reported among 97 hospitals in 2023, for an incidence of 0.14 per 10,000 patient-days. We fitted both a linear regression and an exponential regression model to the infection rates. The exponential model provided a better fit, as evidenced by a higher R^2^ value of 0.76 compared to the linear model’s R^2^ of 0.68. Furthermore, several of the assumptions of linear regression were not met such as linearity and homoscedasticity. Hence, these data indicate that CPE infection rates in Canada have increased exponentially from 2010 to 2023 (0.03 to 0.14 per 10,000 patient days; R^2^ = 0.76, *p* < 0.001) (Fig. 1).

In addition, even though CPE bacteremia rates have significantly increased between 2010 and 2023 (from 0.01 [*n* = 3] to 0.03 [*n* = 31] per 10,000 patient-days, respectively, *p* < 0.001) the magnitude of the increase remains small from a clinical perspective at the national level.

Between 2010 and 2017, regional CPE infection rates were low, however a recent increase has been observed across all three regions. From 2018 to 2023, rates in the Central region tripled (0.04 to 0.13 per 10,000 patient days, *p* = 0.02) and doubled in the Western region (0.07 to 0.15 per 10,000 patient days, *p* = 0.07). CPE infection rates increased in the Eastern region between 2022 and 2023, however this represents few cases (0.01 [*n* = 1] to 0.09 [*n* = 9] per 10,000 patient-days respectively, *p* = 0.03). CPE infection rates were highest in large hospitals (≥ 500 beds) among which the incidence more than tripled from 2020 to 2023 (0.06 to 0.19 per 10,000 patient-days, *p* = 0.04).

In an analysis restricted to 28 hospitals that participated in all surveillance years, we found that the incidence of CPE infections followed a nearly identical trend, except for infection rates in 2023 which were higher (0.17 per 10,000 patient-days) in the restricted analysis compared to the primary analysis of 97 hospitals (0.14 per 10,000 patient-days, *p* = 0.1).

**Fig. 2:**
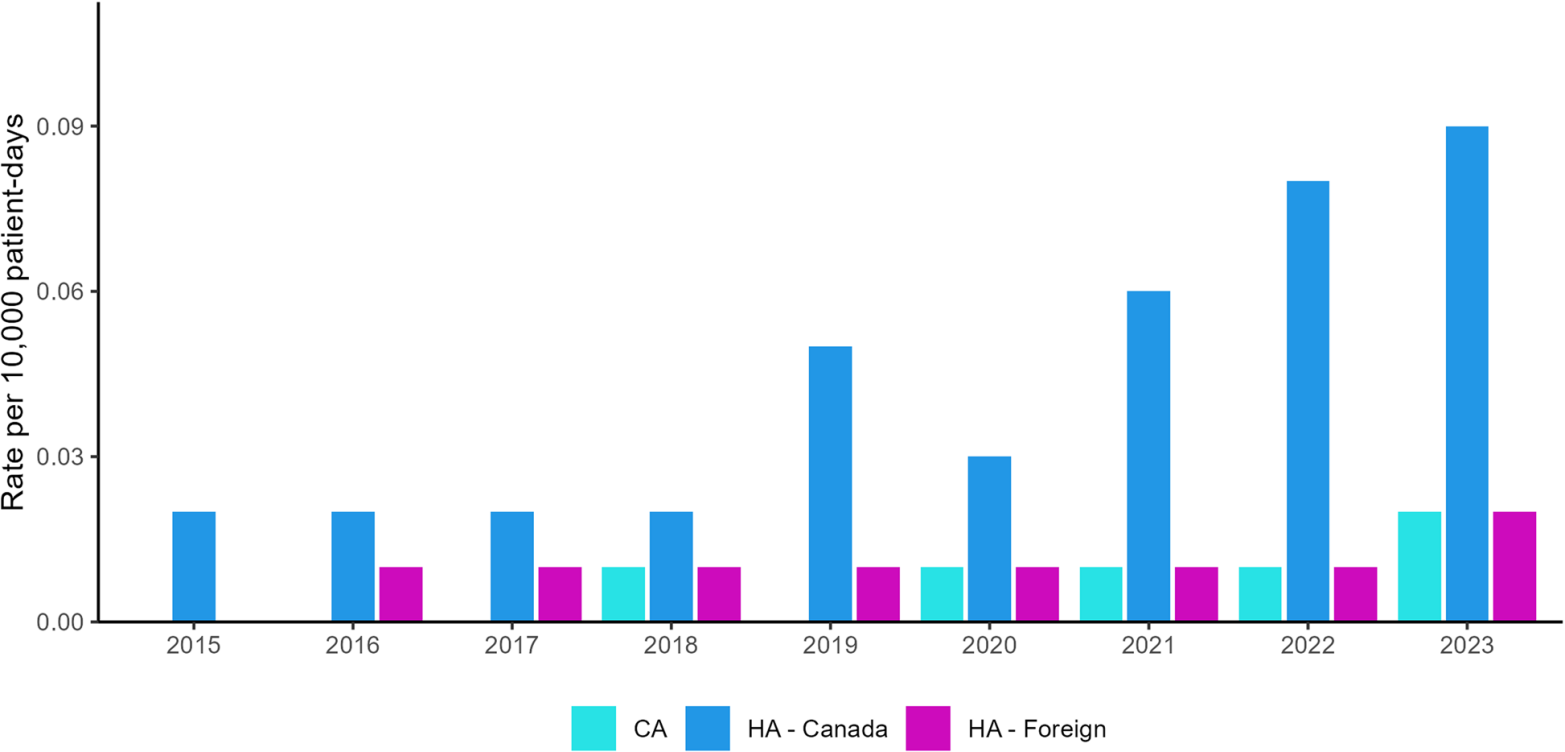
Incidence of CPE infections by acquisition, 2015–2023

The increase in national CPE infection rates coincides with an increase in the rate of HA-CPE infections in Canadian acute care hospitals. The incidence of HA-CPE infection was low and stable from 2015 to 2018 (0.02 to 0.02 per 10,000 patient-days) and significantly increased from 2019 to 2023 (0.05 to 0.09 per 10,000 patient-days, *p* = 0.04), except for a slight decrease in 2020 (0.03 per 10,000 patient-days) (Fig. 2). Similar to overall CPE infection trends, HA-CPE infection rates were highest among hospitals in the Western and Central regions. From 2015 to 2023, the rates of HA (foreign healthcare exposure) CPE infections and community-associated CPE infections remained low with few cases reported (Fig. 2).

**Fig. 3:**
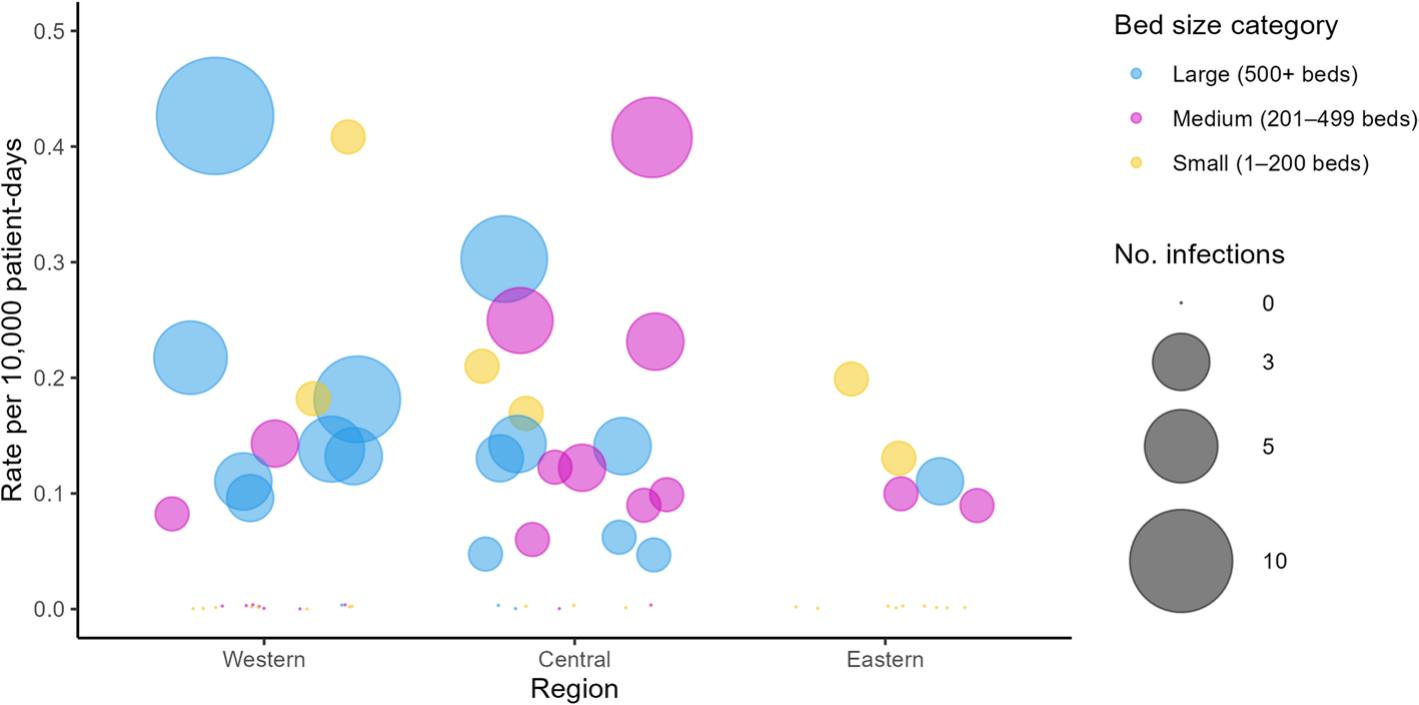
HA-CPE infection hospital rates by region, bed size and number of infections, 2023

Over half of the participating hospitals (66%; 64/97) did not report any HA-CPE infections in 2023, while 7/97 hospitals (7%) accounted for half (53%; 46/87) of all HA-CPE infected cases. This suggests that select hospitals, primarily large hospitals (≥ 500 beds) located in Western and Central Canada, are driving the national incidence rate of HA-CPE infection. Although, there are several small hospitals that report high incidence rates, their impact on the national trend is negligible as their overall number of cases is low. (Fig. 3).

Furthermore, surveillance data indicate an increase in the spread of CPE infections across Canadian hospitals. Among 51 hospitals which participated in surveillance in 2015 and 2023, 16% (8/51) reported at least one HA-CPE infection in 2015 which significantly increased to 41% (21/51) in 2023 (*p* = 0.008).

### CPE colonization results

**Fig. 4:**
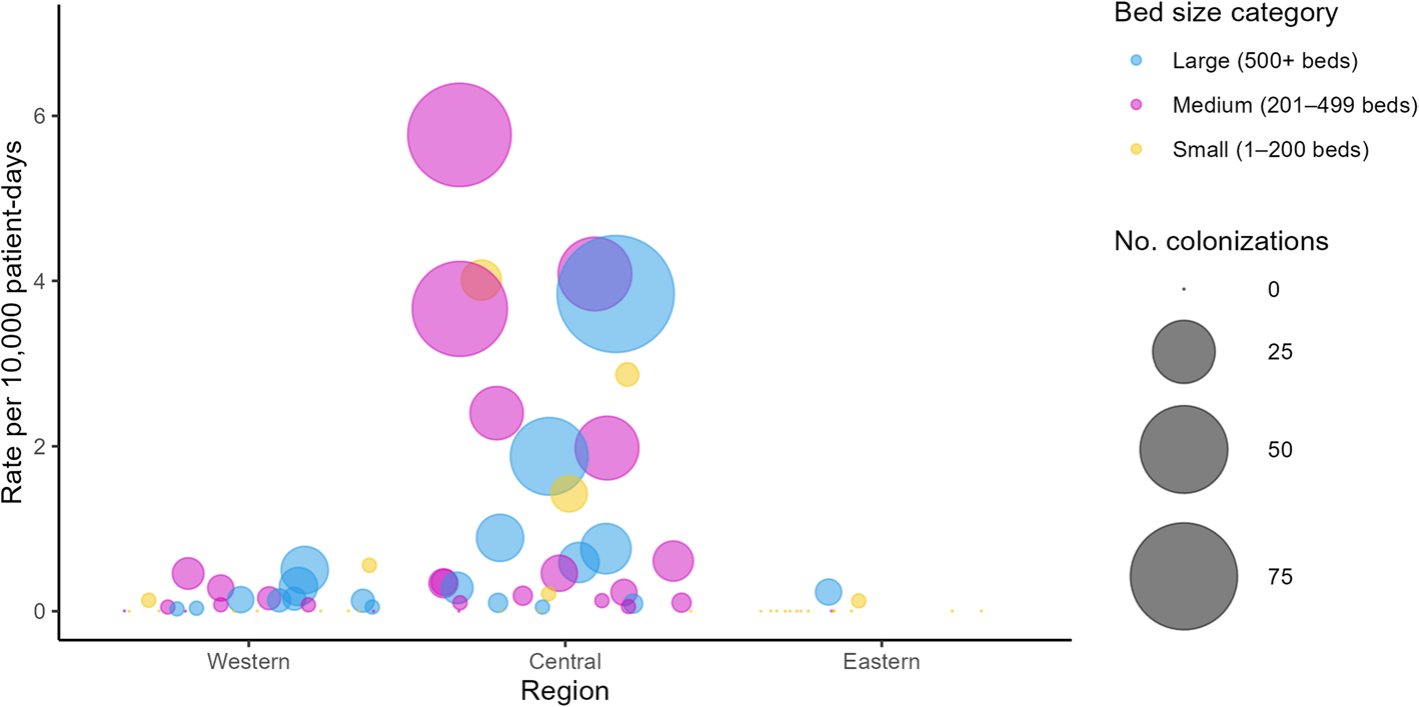
HA-CPE colonization hospital rates by region, bed size and number of infections, 2023

Similarly, we report an exponential increase in CPE colonization rates from 2010 to 2023 (0.02 to 0.78 per 10,000 patient days; R^2^ = 0.83, *p* < 0.001) (Supplemental Figure S1). The increase in colonization rates is largely driven by hospitals in the Central region (0.02 to 1.37 per 10,000 patient-days) compared to Western (0.03 to 0.4 per 10,000 patient-days) and Eastern (0 to 0.08 per 10,000 patient-days) regions. In 2023, HA-CPE colonization rates were highest among medium (201–499 beds) and large (≥ 500 beds) hospitals in the Central region (Fig. 4). Data collected on screening practices among 84 CNISP hospitals identified that in 2023, 35% of hospitals in the Central region (9/26) screened all patients on admission for CPE compared to hospitals in the Western (23%, 8/35) and Eastern (22%, 5/23) regions.

### Microbiology results

Carbapenemases were most frequently detected in *Escherichia coli* (995/3,459, 29%), *Klebsiella pneumoniae* (754/3,459, 22%), and *Enterobacter cloacae* complex (550/3,459, 16%). The most frequently identified carbapenemase families were *bla*_KPC_ (1,326/2,914, 46%), *bla*_NDM_ (842/2,914, 29%) and *bla*_OXA-48_ (462/2,914 16%). There are regional differences in the distribution of resistance genes: the predominant carbapenemase genes identified in the Western region were *bla*_NDM_ (51%), followed by *bla*_OXA-48_ (19%) and *bla*_KPC_ (15%), whereas *bla*_KPC_ predominates in the Central region (56%), followed by *bla*_NDM_ (21%) and *bla*_OXA-48_ (15%). In the Eastern region *bla*_NDM_ represents the majority of cases (71%), followed by *bla*_OXA-48_ (12%) and *bla*_KPC_ (5%).

**Fig. 5:**
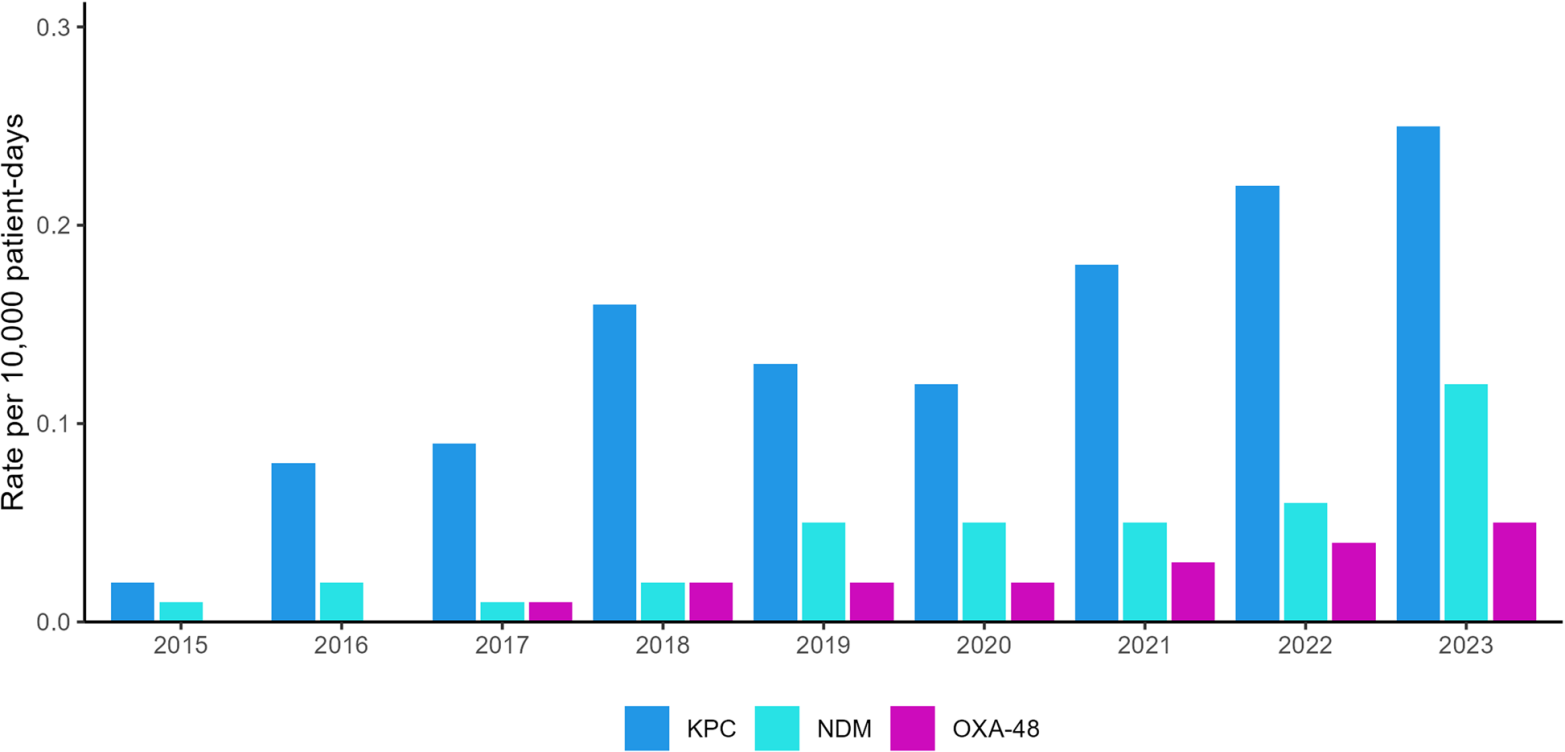
HA-CPE rates by carbapenemase gene, 2015–2023

An increasing trend was observed for HA-CPE *bla*_KPC_ isolates (*p* = 0.001), HA-CPE *bla*_NDM_ isolates (*p* = 0.001) and HA-CPE *bla*_OXA-48_ isolates (*p* < 0.001) from 2015 to 2023 (Fig. 5). However, *bla*_KPC_ was the predominant carbapenemase among all HA-CPE isolates (62%, 950/1,534). Furthermore, international travel was significantly less common among *bla*_KPC_ associated isolates (7.1%) compared to *bla*_OXA-48_ (57%) and *bla*_NDM_ (55%) associated isolates (*p* < 0.001). Receipt of medical care abroad was also significantly less common among *bla*_KPC_ associated isolates (5.3%) compared to *bla*_OXA-48_ (39%) and *bla*_NDM_ (45%) associated isolates (*p* < 0.001).

## Discussion

Surveillance data from a national network of Canadian acute care hospitals indicates an exponential increase in the incidence of CPE infection and colonization from 2010 to 2023. Our findings also show that the rates of HA-CPE colonization and infection are rapidly rising, particularly from 2021 to 2023, suggesting that hospital transmission is driving the recent increase in CPE incidence in Canadian hospitals. Even though the 30-day mortality rate of patients with infection remains lower in Canada (19%) than in other jurisdictions (11%−41%) [31–35], the observed exponential growth is a warning as the current analyses suggest that Canada will continue to see an acceleration in the increase of CPE, with infections forecasted to nearly double by the end of 2025. These findings suggest that current infection control measures in at least some hospitals are insufficient to prevent the transmission of CPE. Potential drivers for the rise in hospital transmission may include inadequate screening strategies, suboptimal application and adherence to barrier precautions, lapses in hand hygiene and environmental cleaning, limited IPAC resources, interfacility patient transfers and under detection of reservoirs not addressed by current IPAC recommendations [2, 5–7, 12, 34, 36–39]. The role of emerging interventions to reduce the spread of CPE, in particular those targeting environmental contamination such as sinks and drains, updating IPAC recommendations to screen patients with exposure to healthcare in Canada and incorporating single patient rooms in hospital construction plans as well as the surveillance of antimicrobial consumption and prudent use of antimicrobials should be further explored [6, 40–47]. In addition, these data highlight the need to ensure timely access to antimicrobials effective for therapy of these infections [48], as well as the critical importance of antimicrobial stewardship programs.

Findings from other Canadian studies also suggest that local acquisition and transmission of CPE is occurring. Kohler et al. [23] found that between 2007 and 2015, 71% of CPE infections were hospital acquired. Several Canadian hospitals have described CPO clusters and outbreaks among patients with no prior history of travel or exposure to healthcare abroad and subsequent nosocomial transmission [19–21].

Results from multiple countries indicate that nosocomial transmission is contributing to the dissemination of CPE. In Europe, widespread dissemination of CPE within healthcare was identified, as 15/31 (48%) countries reported an epidemiological state of either intra-hospital transmission or endemicity in 2018 [9]. Whole genome sequencing and epidemiological data of *K. pneumoniae* isolated from patients in 244 hospitals in 32 European countries found that over half of these hospitals experienced intra-hospital and inter-hospital transmission [49]. Scotland reported an increase in HA-CPO surveillance cultures from 2003 to 2017 as the majority (71% [149/211]) were classified as HA [31]. Grundman et al. [50] identified a clear association between the prevalence of carbapenemase-producing *K. pneumoniae* and *E.coli* with healthcare as most isolates were either acquired in hospital, often associated with intensive care treatment or isolated from patients with a previous hospital admission. Findings from Norway suggest a change in the epidemiology of CPE from sporadic to HA cases as well as clonal outbreaks [10]. Similarly in Belgium, although the initial sporadic occurrence of CPE was reported in patients returning from travel abroad, the majority of CPE patients (70%) did not report a history of travel or receipt of medical care abroad, suggesting local acquisition [4]. Danish national surveillance data show that CPE is spreading in hospitals, with cases rising by 43% from 2022 to 2023. While CPE has historically been linked to travel, domestically acquired cases are increasing globally as well as continued CPE outbreaks despite extensive screening and environmental cleaning measures [13].

The majority of CPE colonized and infected patients (66%) captured in our surveillance did not report travel in the 12 months prior to positive culture and 74% did not report receiving medical care abroad. We observed that travel and receipt of medical care were less commonly reported among *bla*_KPC_ associated cases compared to *bla*_NDM_ and *bla*_OXA-48_ associated cases. This aligns with the higher and increasing HA-CPE rates identified among *bla*_KPC_ associated cases and high HA-CPE rates in the Central region where *bla*_KPC_ is the predominant gene. Furthermore, our surveillance data indicate that select hospitals accounted for most HA-CPE infections. These findings provide further support that local acquisition and outbreaks of CPE in certain regions/hospitals are driving the propagation in Canada. On a positive note, given the limited number of hospitals reporting HA-CPE infections, there is still an opportunity to prevent the widespread dissemination of CPE across Canadian hospitals with vigilant infection control practices and antimicrobial stewardship.

A review of CPE screening practices in Canada highlighted wide regional variation in approaches to screening, which may contribute to the differences in colonization rates reported here [51]. A 2018 survey conducted by Jamal et al. [6] in Ontario, Canada found variability in infection prevention and control practices for CPE, including screening, between hospitals. The observed increase in colonization rates may reflect enhanced routine screening practices in addition to screening in response to local outbreaks and clusters. However, it is important to note that an increase in screening cannot explain the increase in clinical infections.

Our surveillance has several limitations. First, adjudication of acquisition is dependent on the information available in the patient chart and subject to best clinical judgement which may have resulted in the misattribution of cases as healthcare-associated. However, these data were collected by experienced and trained infection control professionals within the CNISP network. Second, our rates reflect CPE cases among admitted patients only. Therefore, patients who tested positive in the emergency ward and later admitted are not included in inpatient rates, which may have underestimated rates. Third, patient-level treatment data were not collected to understand their impact on CPE infection outcomes. Fourth, regional and temporal variations in screening practices likely contributed to the observed differences in CPE colonization rates as well as the patient ward at the time of positive culture. Furthermore, we were unable to assess the contribution of hospital level outbreaks, which limits our ability to interpret these surveillance findings. Fifth, there is the potential for selection bias as hospital participation in the CNISP network is voluntary however, the network represents approximately 37% of acute care beds in Canada and efforts to improve the representativeness of these surveillance data are ongoing [27]. Finally, trends were not reported by province; this was to protect the confidentiality of individual hospitals as some provinces have few participating hospitals.

## Conclusions

We used robust, laboratory and epidemiological linked data collected using standardized definitions from a network of acute care hospitals in Canada to describe novel trends in the national and regional incidence of CPE over a 14-year period. Canadian national surveillance data highlight that the incidence of CPE is increasing at an exponential rate, primarily driven by nosocomial transmission. CPE surveillance within hospital infection control programs is essential for rapid detection to mitigate transmission, guide appropriate antimicrobial therapy and in turn improve patient outcomes. Further investigation, particularly genomic studies including whole genome sequencing, are needed to better understand the transmission routes and most effective control measures to contain the spread of CPE in Canadian hospitals.

## Supplementary Information

Supplementary Material 1.

## Data Availability

The national patient-level dataset analysed during the current study are available from the corresponding author upon reasonable request. The patient-level dataset generated and analysed during the current study are not publicly available due to the binding data sharing agreements with the hospitals involved in the surveillance program.

## Abbreviations

CPE: Carbapenemase-producing *Enterobacterales*
CPO: Carbapenemase-producing organisms
CNISP: Canadian Nosocomial Infection Surveillance Program
PHAC: Public Health Agency of Canada
NML: National Microbiology Laboratory
HA: Healthcare-associated
ICU: Intensive care unit
IQRs: Interquartile ranges
BMT: Bone marrow transplant
SOT: Solid organ transplant
NA: Not applicable
