# Supplementary material for: The evolving epidemiology of Carbapenemase-producing Enterobacterales in Canadian acute care facilities, 2010–2023
Source: Antimicrob Resist Infect Control. 2025 Jul 12;14:88. doi: 10.1186/s13756-025-01602-w (PMC12256002; doi:10.1186/s13756-025-01602-w)
Supplement: Supplementary file 1 — Supplementary Material 1. [file 13756_2025_1602_MOESM1_ESM.docx]

**Supplementary information**

**Table S1. 2023 CPO patient data collection form**

|  | Which laboratory conducted carbapenemase confirmatory testing for this case?  □ NML  □ Provincial laboratory  □ Hospital laboratory | |
| --- | --- | --- |
|  | Is this isolate associated with an infection or a colonization?  **□** Infection^[[1]](#footnote-1)^  □ Colonization | |
|  | CHEC Site: **________________________** | |
|  | Unique Patient Identifier: **________________ YY _________________(**e.g. 99Z23001)  *(CHEC site #) (year) (case number)* | |
|  | Patient ward when positive specimen was collected:  □ Inpatient: If inpatient please check one of the following:  □ ICU  □ NICU  □ Medical ward  □ Surgical ward  □ Other inpatient ward (*specify):_****___________________________________________***  □ Emergency Room (ER)  If the positive specimen was collected while the patient was in ER, was this patient subsequently admitted? □ Yes □ No □ Unknown  □ Outpatient  □ Other ward, please specify:***_________________________________________________________***  □ Unknown | |
|  | Date of birth:  **_____ / _______ / _________**  DD MMM YYYY | Age _______________  □ Years □ Months □ Days |
|  | Sex: □ Male □ Female □ Unknown | |
|  | Date of admission:  **_____ / _______ / _________**  DD MMM YYYY | |
|  | Type of CPO isolate:  **□** Screening isolate  □ Clinical isolate  □ Blood | |
|  | Date of positive culture:  (S*pecimen collection date from which the positive organism was isolated):*  **_____ / _______ / _________**  DD MMM YYYY | |
|  | Organism isolated:  □ Acinetobacter baumannii □ Citrobacter spp.  □ Serratia spp. □ Morganella morganii  □ Klebsiella pneumoniae □ Enterobacter cloacae  □ Enterobacter spp. □ Klebsiella oxytoca  □ Escherichia coli □ Citrobacter freundii  □ Proteus spp □ Serratia marcescens  □ Other, specify: ________________________________ | |
|  | Site of isolation:  (Please select the site of isolation for the isolate that was submitted to the NML)  □ Blood □ Skin/soft tissue  □ Urine □ Stool/rectal swab  □ Wound □ Sputum/Endotracheal secretions/BAL  □ Surgical site □ Other, specify: ________________________________ | |
| 13a. | Where was this CPO acquired?  □ Healthcare-associated – acquired in your acute-care facility (HA-YAF)^[[2]](#footnote-2)^  □ Healthcare-associated – acquired from any other healthcare exposure in Canada (HA-Other, Canada)^[[3]](#footnote-3)^ 🡺 skip to Q14a.  □ Healthcare-associated – acquired from any other healthcare exposure outside of Canada (HA-Other, outside Canada)^[[4]](#footnote-4)^ 🡺 skip to Q14a.  □ Community-associated (CA)^[[5]](#footnote-5)^ 🡺 skip to Q14a.  □ Unable to determine 🡺 skip to Q14a. | |
| 13b. | If healthcare-associated in your facility (HA-YACF), is there evidence of any of the following modes of transmission? Please select all that apply.  □ N/A (not HA-YACF)  □ Sink/drain  □ Hemodialysis  □ Other environment exposure, specify: **_____________________________________________**  □ Device/procedure (e.g. ERCP, endoscopy), specify:**____________________________________**  □ Another patient (e.g. contact tracing, outbreak investigation).  If possible, please specify the PID: **___________________________________________________**  □ Other exposure. Specify: **________________________________________________________**  □ Unknown | |
| 14a | Is there any evidence of international travel in the 12 months prior to the patient’s CPO diagnosis?  □ No, there is no evidence of international travel. 🡺 if NO, skip to Q15.  □ Yes, specify where travelled to: **____________________________________**  □ Unable to determine | |
| 14b. | If traveled internationally, is there evidence the patient received medical care where they traveled  to?  □ N/A - no evidence of international travel  □ Yes, there is evidence that the patient sought medical care while on international travel  □ No, there is no evidence that the patient sought medical care while on international travel  □ Unable to determine | |
| 15. | Is there any evidence of international travel by a member of the household or caregiver in the 12  months prior to the patient’s CPO diagnosis?  □ No, there is no evidence of international travel.  □ Yes, specify where travelled to: **____________________________________**  □ Unable to determine | |
| 16 | Is there evidence the patient has pre-existing comorbidities(s)? Please check all that apply.  □ No evidence of any pre-existing comorbidity  □ Yes *(please check all that apply)*  □ Diabetes  □ Liver disease  □ HIV infection  □ Cancer (active)  □ Lung disease (e.g., asthma, COPD)  □ Kidney disease (include all patients on dialysis)  □ Solid organ transplant recipient  □ Bone marrow transplant recipient  □ Other immunosuppression, specify _________  □ Heart disease  □ Other, specify _________________________  □ Unknown | |
| 17 | During this admission or in the 14 days prior to this admission, did this patient test COVID-19 positive for the first time?  □ Yes - if your site participates in VRI surveillance, please provide the PID for the COVID-19 patient questionnaire: __________  □ No  □ Unknown | |
| **Q18 and Q19 are only to be completed for infected cases** | | |
| 18 | Was the patient admitted to an ICU within 30 days of positive culture?  □ N/A - patient was already in an ICU at the time the positive culture was obtained  □ Yes, please indicate the date of ICU admission: **_____ / _______ / _________**  DD MMM YYYY  □ No  □ Unknown | |
| 19 | What was the patient outcome 30 days after positive culture?  □ Patient alive, still in hospital  □ Patient survived and discharged  Date of discharge **_____ / _______ / _________**  DD MMM YYYY  □ Patient survived and transferred  Date of transfer **_____ / _______ / _________**  DD MMM YYYY  □ Patient died  Date of death **_____ / _______ / _________**  DD MMM YYYY  □ Unknown | |
| 20 | If the patient died within 30 days after the positive culture, please indicate the relationship of CPO to the death  □ CPO was the cause of death  □ CPO contributed to death  □ Death is unrelated to CPO  □ Causality between CPO and death cannot be determined | |

**Supplementary Table S2. Selected characteristics of hospitals participating in CPE surveillance, 2010 and 2023**

| **Characteristic** | **2010 N=30^1^** | **2023 N=97^1^** | **p-value** |
| --- | --- | --- | --- |
| **Region** |  |  | **0.016^2^** |
| Central | 19/30 (63%) | 33/97 (34%) |  |
| Western | 6/30 (20%) | 39/97 (40%) |  |
| Eastern | 5/30 (17%) | 25/97 (26%) |  |
| **Hospital bed size category** |  |  | 0.66^2^ |
| Small (1–200 beds) | 11/30 (37%) | 44/97 (45%) |  |
| Medium (201–499 beds) | 13/30 (43%) | 34/97 (35%) |  |
| Large (500+ beds) | 6/30 (20%) | 19/97 (20%) |  |
| **Teaching hospital** | 29/30 (97%) | 62/97 (64%) | **<0.001^2^** |
| **Hospital type** |  |  | 0.11^2^ |
| Adult | 15/30 (50%) | 57/97 (59%) |  |
| Mixed | 6/30 (20%) | 27/97 (28%) |  |
| Pediatric | 9/30 (30%) | 13/97 (13%) |  |
| ^1^n/N (%) | | | |
| ^2^Pearson's Chi-squared test | | | |

**Supplemental Figure S1. Incidence of national CPE colonization rates with 95% confidence intervals and expressed as an exponential equation, 2010-2023**


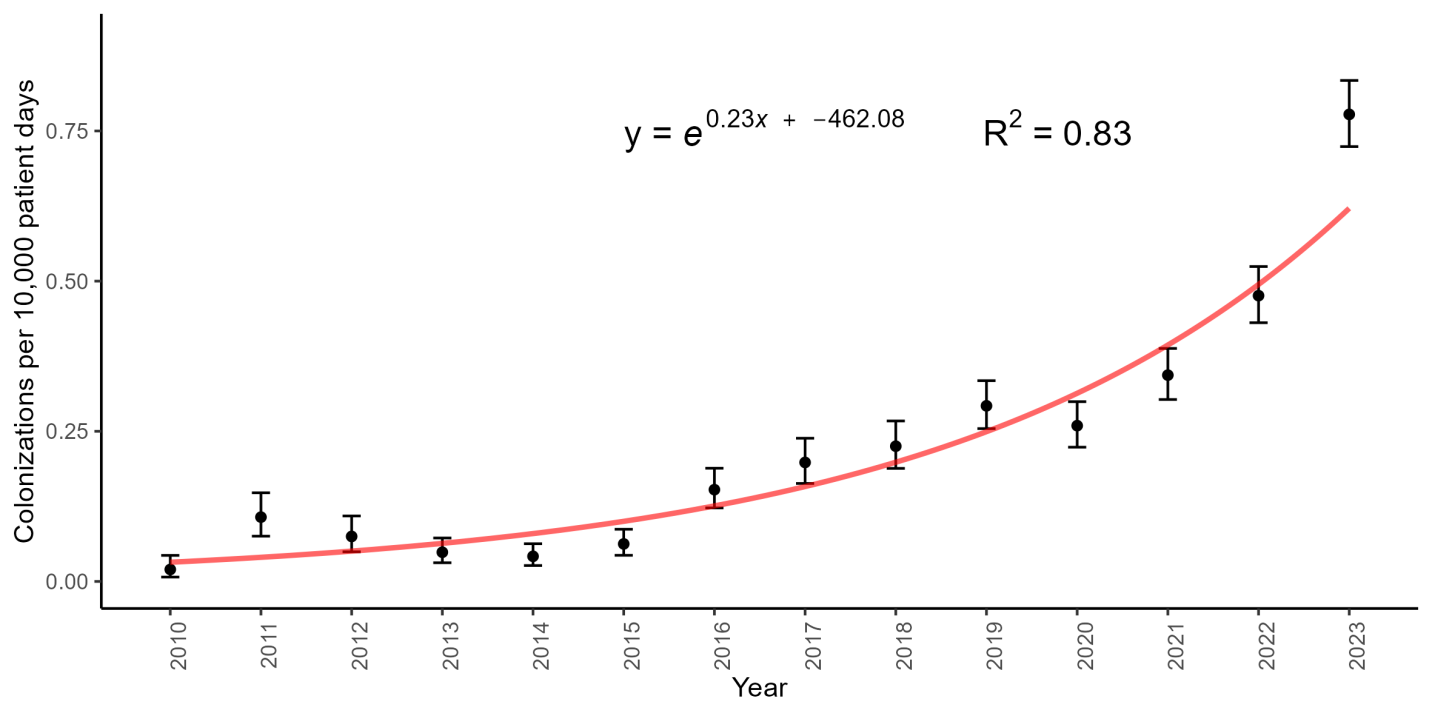


We fitted both a linear regression and an exponential regression model to the colonization rates. The exponential model provided a better fit, as evidenced by a higher R² value of 0.83 compared to the linear model R² of 0.73. In addition, several of the assumptions of linear regression were not met such as linearity and homoscedasticity. We report an exponential increase in CPE colonization rates from 2010 to 2023 (0.02 to 0.78 per 10,000 patient days; R² = 0.83, *p*<0.001). In an analysis restricted to 28 hospitals that participated in all surveillance years, we found that the incidence of CPE colonization followed a similar trend from 2010 to 2023 (0.02 to 1.07 per 10,000 patient-days) compared to rates reported for all participating hospitals from 2010 to 2023 (0.02 to 0.78 per 10,000 patient-days).

**Supplemental Figure S2. Incidence of CPE colonization by acquisition, 2015-2023**

**
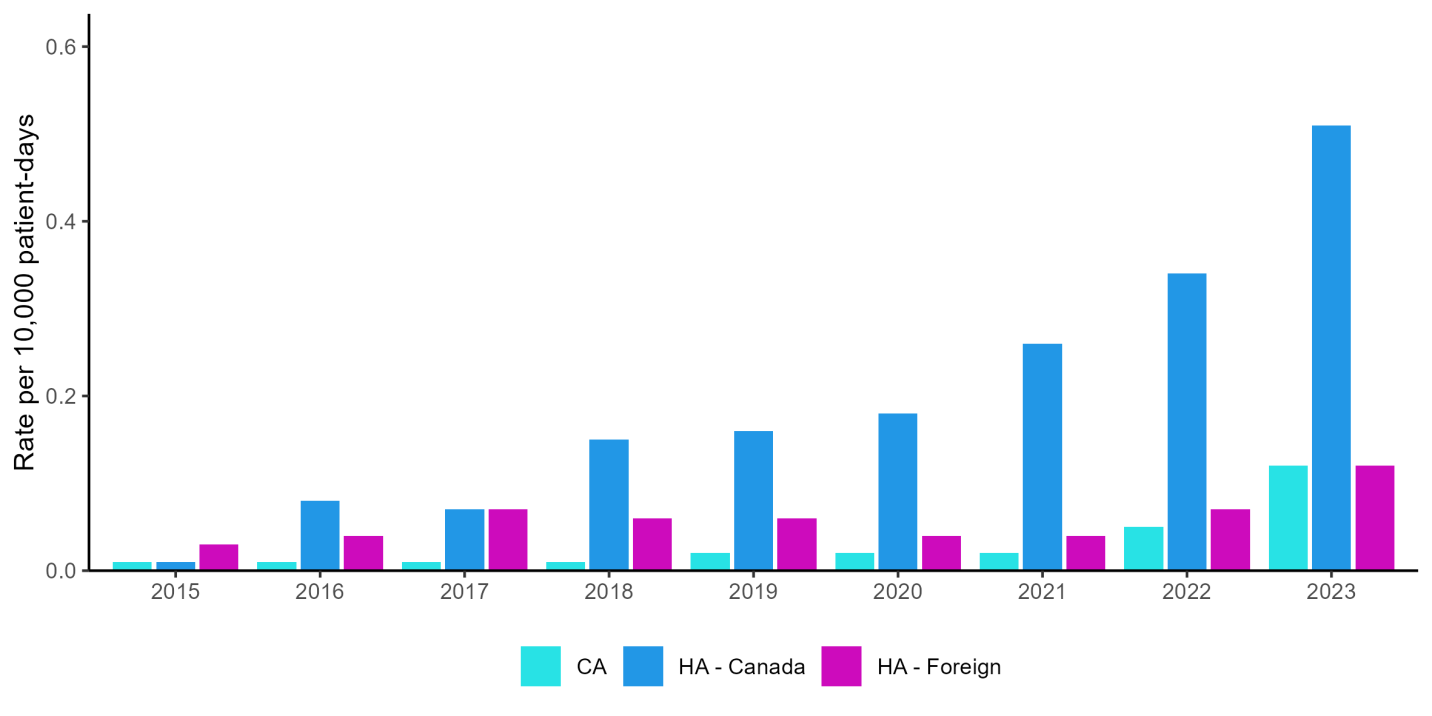
**

While the rates of community-associated and foreign HA CPE colonization increased from 2015 to 2023 (0.01 to 0.12 per 10,000 patient-days, *p*=0.002 and 0.03 to 0.12 per 10,000 patient-days respectively, *p*=0.07), the number of cases and rates remain low. However, the increase in the national incidence of HA-CPE colonization acquired in Canadian facilities from 2015 to 2023 (0.01 to 0.51 per 10,000 patient-days, *p*<0.001) appears to be the major contributor to the increase in overall colonization rates.

1. Infection is determined using the 2023 CDC/NHSN surveillance definitions for specific infections, and in accordance with the best judgment of the healthcare practitioner. These criteria can be accessed at

   [www.cdc.gov/nhsn/PDFs/pscManual/17pscNosInfDef_current.pdf](http://www.cdc.gov/nhsn/PDFs/pscManual/17pscNosInfDef_current.pdf) [↑](#footnote-ref-1)
2. Patient is on or beyond calendar day 3 of their hospitalization OR has had a healthcare exposure (inpatient or outpatient) at your facility that would have resulted in this infection or colonization (using best clinical judgement) [↑](#footnote-ref-2)
3. Any patient who has an infection or colonization not acquired at your facility that is thought to be associated with another healthcare exposure in Canada (e.g. another acute-care facility, long-term care, rehabilitation facility, clinic or exposure to a medical device). [↑](#footnote-ref-3)
4. Any patient who has an infection or colonization not acquired at your facility that is thought to be associated with another healthcare exposure outside of Canada (e.g. another acute-care facility, long-term care, rehabilitation facility, clinic or exposure to a medical device). [↑](#footnote-ref-4)
5. No exposure to healthcare that would have resulted in this infection or colonization (using best clinical judgement) and does not meet the criteria for a healthcare-associated infection or colonization. [↑](#footnote-ref-5)
